# Sucrose-templated interconnected meso/macro-porous 2D symmetric graphitic carbon networks as supports for α-Fe_2_O_3_ towards improved supercapacitive behavior†

**DOI:** 10.1039/d0ra02056g

**Published:** 2020-04-21

**Authors:** Jacob Otabil Bonsu, Jeong In Han

**Affiliations:** Department of Energy and Materials Engineering, Dongguk University – Seoul Pil-dong, Jung-gu 04620 Seoul South Korea; Department of Chemical and Biochemical Engineering, Dongguk University – Seoul 04620 South Korea

## Abstract

In this study, ultrahigh electrochemical performance for interconnected meso/macro-porous 2D C@α-Fe_2_O_3_ synthesized *via* sucrose-assisted microwave combustion is demonstrated. Hematite (α-Fe_2_O_3_) synthesized *via* the same approach gave an encouraging electrochemical performance close to its theoretical value, justifying its consideration as a potential supercapacitor electrode material; nonetheless, its specific capacitance was still low. The pore size distribution as well as the specific surface of bare α-Fe_2_O_3_ improved from 145 m^2^ g^−1^ to 297.3 m^2^ g^−1^ after it was coated with sucrose, which was endowed with ordered symmetric single-layer graphene (2D graphene). Accordingly, the optimized hematite material (2D C@α-Fe_2_O_3_) showed a specific capacitance of 1876.7 F g^−1^ at a current density of 1 A g^−1^ and capacity retention of 95.9% after 4000 cycles. Moreover, the material exhibited an ultrahigh energy density of 93.8 W h kg^−1^ at a power density of 150 W kg^−1^. The synergistic effect created by carbon-coating α-Fe_2_O_3_ resulted in modest electrochemical performance owing to extremely low charge transfer resistance at the electrode–electrolyte interface with many active sites for ionic reactions and efficient diffusion process. This 2D C@α-Fe_2_O_3_ electrode material has the capacity to develop into a cost-effective and stable electrode for future high-energy-capacity supercapacitors.

## Abstract 2

In this study, ultrahigh electrochemical performance for interconnected meso/macro-porous 2D C@α-Fe_2_O_3_ synthesized *via* sucrose-assisted microwave combustion is demonstrated.

## Graphical abstract

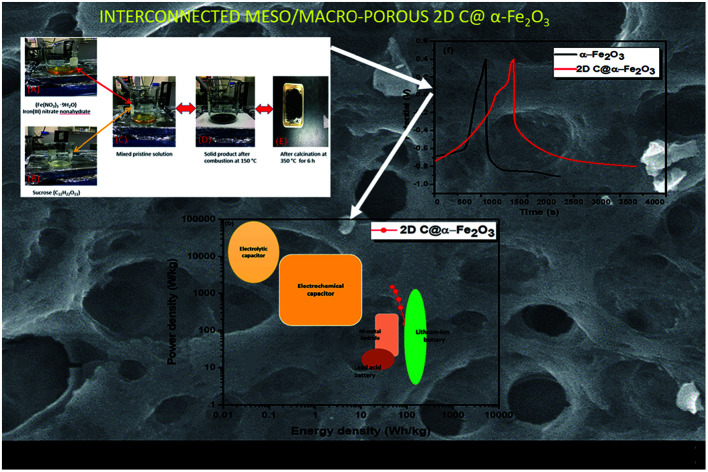

## 1. Introduction

With the exponential growth of technology and increase in energy crises in the 21st century with respect to the numerous energy generation approaches, insistence for green and effective energy production has become a global concern. For the past few years, supercapacitors (electrochemical capacitors) have attracted considerable attention for applications spanning from handy electronic devices up to hybrid electric vehicles owing to their rectitude of exceptional power performance, long cycle span, rapid charging–discharging rate, environmental friendliness and wide temperature range.^1–4^ Supercapacitors can be categorized precisely into two main operating models, namely, pseudocapacitors and electric double-layer capacitors (EDLCs), which are classified primarily based on their energy storage principles. Pseudocapacitors store energy by rapid faradaic chemical reactions, while EDLCs base their storage mechanism on the absorption of ionic species at the electrode–electrolyte interface.^5^ Supercapacitors perform well in relation to their power density, but several drawbacks crop up when it comes to energy density, in which batteries possess a sturdy advantage. Techniques for enhancing the supercapacitor performance have typically been pivoted on developing unique electrode materials that can generate very good specific capacitance and making use of pertinent electrolytes that scan to a vast potential window.^6^ Numerous electrode materials have been analysed for developing supercapacitors with the aim of improving electrochemical performance. Among them, transition metal oxides happen to be one of the materials that are frequently studied, and this is ascribed to their rapid and effective faradaic chemical reactions.^7^ For example, pervasive analyses have been conducted with different metal oxides including NiO,^8^ RuO_2_,^9^ SnO_2_,^10^ Fe_2_O_3_,^11^ MnO_2_,^12^ and Co_3_O_4_ (ref. 13) to improve the specific capacitance and energy density of supercapacitors. Altogether, oxides of iron, not to mention maghemite (γ-Fe_2_O_3_), hematite (α-Fe_2_O_3_) and magnetite (Fe_3_O_4_), retain a large theoretical specific capacitance of 924 mA h g^−1^ for magnetite and 1007 mA h g^−1^ for hematite.^14^ This makes them very promising electrode materials for fabricating efficient electrochemical energy conversion and storage devices. Among these iron metal oxides, hematite has been exhaustively studied by virtue of its low toxicity, natural abundance, low cost, environmental friendliness and high theoretical capacity.^15^ The reversible reduction/oxidation between Fe^2+^ and Fe^3+^ accounts for the pseudocapacitive performance of hematite;^16^ nonetheless, the low surface area, poor conductivity and bad cycle stability of hematite hinders its application as an effective electrode material.^17^ An appropriate scheme to curb these drawbacks is to desegregate it with conductive carbon-containing materials such as carbon nanotubes,^18^ carbon black,^19^ sucrose,^20^ graphenes,^21^ carbon aerogels,^22–26^ and activated carbon.^27,28^ Many factors such as the precursor used, preparation method and calcination temperature could influence the property and morphology of the resulting carbon-coated material.^29^ This aids in creating a synergistic effect of preventing particle agglomeration and sintering, which improves the porosity of the composite.^30,31^ Sucrose particularly is very attractive to form composites with hematite due to its relatively high chemical purity and uniform structure.^20^

Sucrose has been widely used to synthesize mesoporous and macroporous carbon by the template method. Carbon derived from sucrose can attain a high specific surface area of 1941 m^2^ g^−1^ and relatively high specific capacitance.^32^ Microporous, macroporous and mesoporous carbon structures have been widely studied for various energy storage systems including supercapacitors.^33,34^ Porous carbon structures endowed with hierarchical macro–meso–micro porosities promote fast ion transport that minimizes the diffusion distance between the electrode and electrolytes and also reduces the volume change during the charge/discharge cycling, which primarily improves supercapacitor performance.^35–37^ However, a cost effective and green method to synthesize porous carbon materials with tunable pore sizes still poses an enormous challenge.^38^ There have been several reports on sucrose being employed to composites or to enhance the performance of supercapacitor electrode materials, but to the best of our knowledge, no report has been published in light of this 2D C@α-Fe_2_O_3_ electrode material's synthesis scheme and electrochemical performance.

The synthesis process originates from mixing metal nitrates and organic fuels such as sucrose, citric acid, and urea. With all the afore-mentioned organic fuels, sucrose presents itself as the cheapest, nontoxic, facilely acquired at industrial scale and easy to handle and reserve at low temperatures. Microwave combustion synthesis route employed in the process is environmentally friendly and facile, and it prevents chemical wastage and takes very little time for the process to be done.

Herein, we report a consequential sucrose-assisted microwave combustion synthesis method to develop a network of interconnected meso/macro-porous structures from a blend of sucrose and hematite. The synergistic effect of the two electrode materials gave rise to an ordered symmetric single-layer graphene structure, which contributed immensely to the exceptional electrochemical performance. This exceptional morphology gave an ultrahigh specific capacitance and energy density comparatively with very good mechanical stability after a reasonable cycle runtime.

## 2. Experimental section

### 2.1. Materials and reagents

All chemicals and reagents employed in this research were procured from Sigma-Aldrich Chemical Reagent Co., Ltd and Guangzhou Chemical Reagent Company. They were of analytical grade, and were used without further purification.

### 2.2. Synthesis of samples

In an illustrative synthesis, a network of interconnected meso/macro-porous 2D C@α-Fe_2_O_3_ composites were synthesized *via* a sucrose-assisted microwave combustion synthesis route. Primarily, 0.808 g of Fe(NO_3_)_3_·9H_2_O (2 mmol) precursor and 0.684 g of sucrose (C_12_H_22_O_11_, 2 mmol) were dissolved in a beaker each with 3 mL of deionized water respectively under constant magnetic stirring for 15 minutes. The respective solutions undergo rapid ultrasonication for 10 minutes and then got mixed slowly under constant magnetic stirring for 30 minutes to form a homogenous mixed solution. The beaker which contains 6 mL of the apparent resulting solution was placed in a preheated convectional microwave, heated up to a temperature of 150 °C and maintained for 1 h. The beaker is then naturally cooled to room temperature, and the resulting solid product is grinded into a loose powder. The sample was calcined in a furnace at 350 °C at a heating rate of 5 °C min^−1^ and held for 6 h in a nitrogen atmosphere. The obtained final products were defined as meso/macro-porous 2D C@α-Fe_2_O_3_ composites. α-Fe_2_O_3_ particles were synthesized *via* the same approach with sucrose pretermitted from the synthesis procedure.

### 2.3. Characterization

The microstructure and morphology of the samples were studied using a scanning electron microscope (SEM) (XL30 FEG-SEM Philips), operating at an acceleration voltage of 10 kV, and transmission electron microscopy (TEM) was carried out using a JEOL TEM-2100 electron microscope that worked at 200 kV. The crystal structure and phase composition of the samples were obtained by powder X-ray diffraction (XRD) measurement carried out using a Rigaku D/Max-2500 diffractometer with Cu Kα radiation (40 kV, 30 mA) at a scan rate of 2° min^−1^ between 10° and 80° (2*θ*). Raman spectra were studied using high-resolution (LabRAM) Raman spectrometer with laser excitation at 514.5 nm under ambient conditions. An AXIS HIS 165 spectrometer (Kratos Analytical) was used to run X-ray photoelectron spectroscopy (XPS) equipped with a monochromatized Al Kα X-ray source (1486.71 eV photons) to test the composition of samples. Thermogravimetric (TG) analysis (Netzsch-STA 449C) was conducted from room temperature to 900 °C operating at a constant heating rate of 10 °C min^−1^ in air. Micromeritics ASAP 2020 device was used to conduct nitrogen adsorption–desorption analysis, which helped to determine the porous structure of the sample by the Barrett–Joyner–Halenda (BJH) method. The surface area of the samples was calculated by the Brunauer–Emmett–Teller (BET) method based on the adsorption data in the relative pressure (*P*/*P*_0_) range of 0.05 to 0.3. The overall pore volume was calculated from the amount of nitrogen adsorbed at a relative pressure (*P*/*P*_0_) of 0.99.

### 2.4. Device assembly

The working electrodes of the supercapacitor were prepared by mixing the active material, conductive carbon black and polyvinylidene fluoride (PVDF) in an *n*-methyl-2-pyrrolidone (NMP) solvent in the proportion of 80 : 10 : 10 to form a homogenous slurry. The slurry subsequently was brush-coated onto an already washed (by sonicating in acetone, isopropyl alcohol (IPA) and distilled water, 10 minutes each) nickel foam current collector (1 × 1 cm^2^) and dried at 80 °C overnight. Each electrode had a mass loading of around 2 mg, and the mass of the active material was derived by the difference between the weight of the bare nickel foam and that of the active material coated in that order.

### 2.5. Electrochemical characterization

A typical three-electrode system that is very efficient and systematized for studying the electrochemical-specific characteristics of electrode materials was introduced to investigate the electrochemical behaviour of the samples.^39^ A platinum wire was used as the counter electrode, Ag AgCl^−1^ as the reference electrode and 6 M KOH aqueous solution as an electrolyte for the setup. The galvanometric charge–discharge (GCD) and cyclic voltammetry (CV) measurements were performed on a Biologic SP-150 electrochemical workstation. The cyclic voltammetry (CV) measurement was conducted within a potential window of −0.8 to 0.4 V at different sweeping rates and GCD analyses was equally carried out within the same potential range at different current densities. The cycle performance was studied between −0.8 V and 0.4 V, and electrochemical impedance spectroscopy (EIS) spectra were recorded from 0.01 Hz to 100 kHz and was measured by applying an AC amplitude of 5 mV at open circuit potential.

Specific capacitance *C*_S_ (F g^−1^) was calculated from the galvanometric charge–discharge curve according to the following equation:1
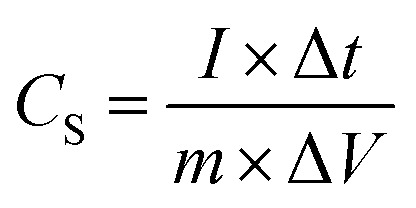
where *I* is the constant applied current (A), the derivative of Δ*t* and Δ*V* (V s^−1^) represents the slope obtained by fitting a straight line to the discharge curve and *m* (g) is the mass of the active materials.

The energy density *E* (W h kg^−1^) can be estimated using the following formula:2
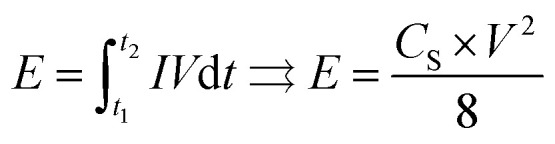
where *V* (V) is the voltage, *t*_1_ and *t*_2_ are the start and the end time in the discharge process respectively and *I* is the constant current density (A g^−1^). The resulting energy equation on the right was derived from numerically integrating the *t*–*V* graph (or *Q*–*V* graph, as the current density *I* is a constant value) area during the discharge process.^40^

Effective series resistance (ESR) was calculated using the drop in voltage at the commencement of the discharge, at constant current.3
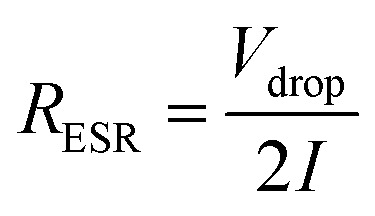


The power density values *P* (W kg^−1^) typically can be calculated from the discharger data at certain constant current I and normalized with the weight of the cell (two electrodes). This is given by,4
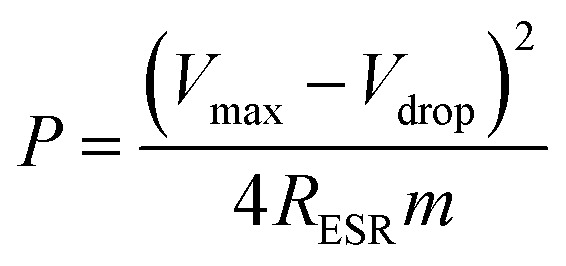


## 3. Results and discussion

### 3.1. Phase composition and morphology of the samples

**Fig. 1:**
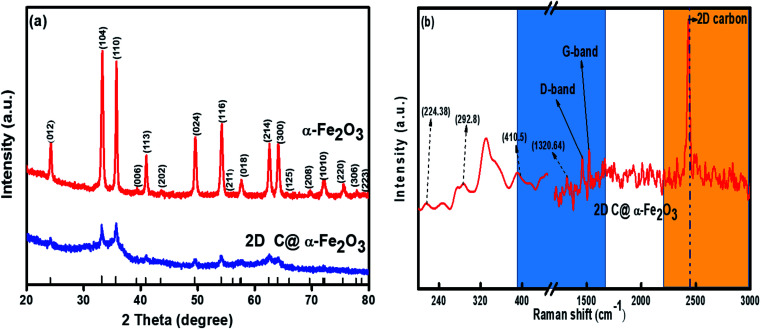
(a) XRD plots of α-Fe_2_O_3_ and 2D C@α-Fe_2_O_3_ and (b) Raman plot of 2D C@α-Fe_2_O_3_.

X-ray diffraction (XRD) profile that primarily helps identify the phase composition of the prepared 2D C@α-Fe_2_O_3_ composite and bare α-Fe_2_O_3_ as contrast is shown in Fig. 1a. It could be clearly observed that the samples depict planar peaks of (012), (104), (110), (006), (113), (202), and (024), respectively in that order as shown. The entire planar peaks of the XRD spectra can be assigned mainly to α-Fe_2_O_3_ with JCPDS no. 33-0664. It could be observed that the peaks of bare hexagonal α-Fe_2_O_3_ are at the same position but more crystalline than those of 2D C@α-Fe_2_O_3_, and this is due to the presence of the carbon in sucrose, and it portrays itself with a very weak hump in the 2 theta range of 43.7 to 43.8 and 22.7 to 22.8 with planes of (101) and (002) respectively. To further study and confirm vehemently the carbon behavior and presence in the product, Raman spectroscopy is carried out to ascertain the vibrational peak mode of the materials (Fig. 1b) and also identify the respective number of layers stacked up in the sample with their physical properties and phenomena.^41^ Bands detected at 224.38, 292.8, 410.5 and 1320.64 cm^−1^ can be assigned as the vibrational peaks of α-Fe_2_O_3_ and in accordance with its formative corresponding peaks.^7^ Allotropes of carbon observed in disordered and graphitic structural form in the sample are examined at peaks 1348 and 1591.5 cm^−1^ with fairly low intensities shown as D and G bands respectively in that order. The disordered D band arises as a result of hybridized vibrational modes associated with graphitic edges in the form of defects in the sample, while the G band is ascribed to the C–C bond stretching vibration in graphitic materials, which is commonly associated with sp^2^ hybridized carbon systems.^42^ The defect concentration is usually measured by the ratio between the intensities of these two bands (*I*_D_/*I*_G_).^43^ A sharp symmetric prominent peak is located at 2434.9 cm^−1^, which is ascribed to the 2D band. The typical 2D band is resonant, and therefore, it depicts the very strong dispersive nature that explains the shift in position and the shape observed. The ratio of the 2D and G bands (*I*_2D_/*I*_G_) was found to be approximately 2, which coupled with a low D band intensity and a very sharp symmetric 2D band confirms a high-quality almost defective free single-layer graphene structure in the product and, accordingly, greatly contributes to the high performance of the material.^44^

**Fig. 2:**
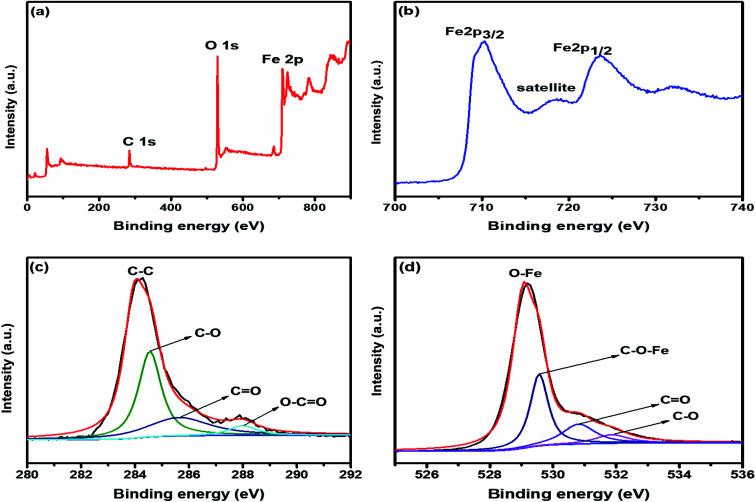
(a) XPS survey spectrum of 2D C@α-Fe_2_O_3_. XPS core level spectrum of (b) Fe 2p; (c) C 1s; and (d) O 1s.

The electronic configuration and composition of the sample were measured using an X-ray photoelectron spectrometer (XPS). As demonstrated in Fig. 2a, three sturdy signals were seen in the full spectrum of 2D C@α-Fe_2_O_3_, representing Fe, C and O elements with the corresponding peaks of O 1s and C 1s at 530.2 eV and 284.6 eV, respectively. A precise and high-resolution XPS spectrum of Fe 2p is shown in Fig. 2b, which depicts two distinct peaks at approximately 712.7 and 725.8 eV correlating with the Fe 2p_1/2_ and Fe 2p_3/2_ electronic orbital state. Additionally, a shake-up satellite peak is seen at a binding energy of 718.4 eV, which confirms the formation of α-Fe_2_O_3._^45^Fig. 2c demonstrates the spectrum of C 1s, which showed high-resolution peaks. It could be observed that there were four kinds of carbon-associated functional groups present in the configuration. A prominent peak centered at approximately 284.9 eV represents the sp^2^ C–C bond.^46^ The peaks located at 285.9, 286.8 and 288.7 are attributed to the C–O bond in alkoxy and epoxy groups, the C=O bond found in carbonyl groups and lastly O–C=O in carboxyl groups.^47^ The lower intensities evidently observed at the bands of C=O and C–O indicate that the oxygen functional groups attached to the carbon structures have been greatly reduced during the calcination process for carbon coating. Fig. 2d also reveals the high resolution of the O 1s spectrum with its major peaks located at 529.6 eV. The peak at the location is ascribed to the Fe–O bond in hematite (α-Fe_2_O_3_).^48^ Other peaks located at 531.1 and 532.2 eV are mapped to surface oxygen–associated groups for C=O and C–O bonds, respectively.^49^ There is a prominent band at 530.1 eV, which is assigned to the Fe–O–C bonds. This proves that hematite is vehemently bonded to the surface of the low-defect single-layer graphitic carbon structure found in sucrose by combining the surface oxygen-associated groups with the Fe^3+^ ion.^50^

**Fig. 3:**
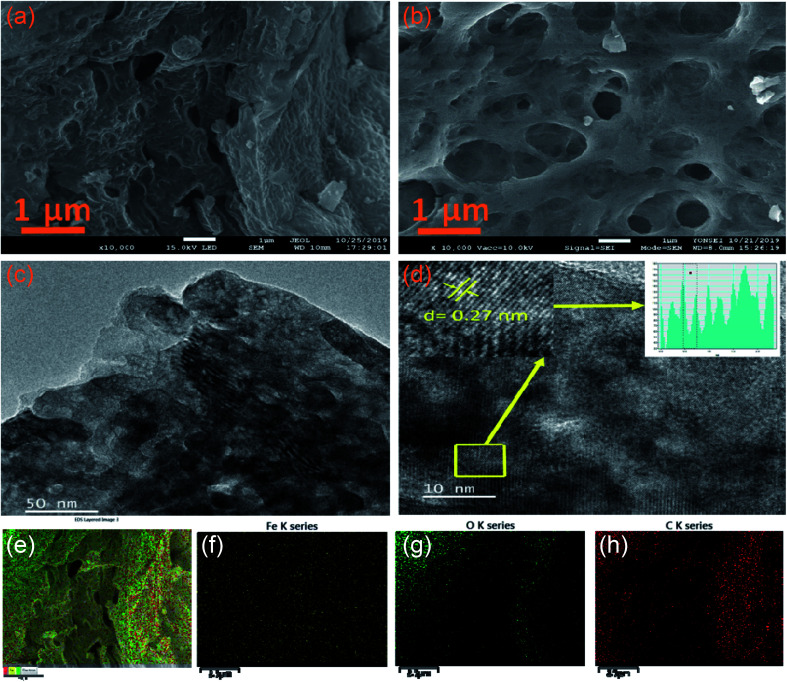
SEM and TEM images of samples: SEM images of (a) α-Fe_2_O_3_ and (b) 2D C@α-Fe_2_O_3_. (c) TEM and (d) HRTEM images of 2D C@α-Fe_2_O_3_. (e) EDS layered image showing the uniform distribution of Fe, O and C. EDS distribution mapping of: (f) Fe, (g) O and (h) C.

The morphological features of the synthesised samples are investigated by scanning electron microscopy (SEM) and transmission electron microscopy (TEM). Fig. 3a and b reveals the SEM images of pristine α-Fe_2_O_3_ and 2D C@α-Fe_2_O_3_, respectively. It could be seen that 2D C@α-Fe_2_O_3_ has a network of interconnected macro- and mesopores, which are numerous and spread out in the material, whereas α-Fe_2_O_3_ has very little and uneven pore distribution across the material. The construction of the morphology observed in 2D C@α-Fe_2_O_3_ is in such a way that the surface of the material is mainly made up of macropores and connected to the internal mesopores facilitating fast charge transfer and mass transport, which is a very exceptional feature of this material. The well-ordered mesopores had an average diameter of 40 nm and macropores had an average diameter of 110 nm compared to those of parent materials, namely, 20 and 70 nm, respectively. To further ascertain the elemental composition of the working electrode material, the EDS mapping is carried out as depicted in Fig. 3e–h. The results gathered from the EDS spectra proved the existence of C, O and Fe elements in the product as shown, which were uniformly dispersed throughout, indicating that homogeneity is attained.

Fig. 3c and d shows the TEM analyses of interconnected macro- and mesoporous 2D C@α-Fe_2_O_3_. The image vividly shows the porous state of the material after it has been coated with carbon, which is ordered and defined. High-resolution TEM image reveals an interplanar *d*-spacing of 0.27 nm fitting to the well-aligned lattice fringes, and this tallies directly to the presence of hematite in the working electrode.

**Fig. 4:**
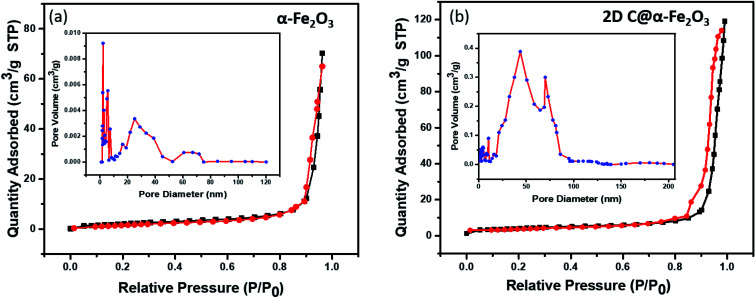
N_2_ adsorption–desorption isotherm and pore size distributions of (a) α-Fe_2_O_3_ and (b) 2D C@α-Fe_2_O_3_.

In Fig. 4, the pore structure and specific surface area of the samples are evaluated as shown. The BJH method was used to analyse the pore size distribution and BET method subsequently used to calculate the specific surface area. It is very obvious that both 2D C@α-Fe_2_O_3_ (Fig. 4b) and α-Fe_2_O_3_ (Fig. 4a) exhibit a type (IV) isothermal plot, showing a clear H3 hysteresis curve which affirms the presence of mesoporous structures of the two products. The specific surface areas were calculated and values of 145 m^2^ g^−1^ and 297.3 m^2^ g^−1^ were obtained for α-Fe_2_O_3_ and 2D C@α-Fe_2_O_3_ respectively. Obviously a comparative analysis of the two samples showed a greater surface area for 2D C@α-Fe_2_O_3_, and this facilitates more active sites which in conjunction with the mesoporous structure can constructively limit the volume alteration of electrode materials in the course of charge–discharge process.^7^ Additionally, this distinctive structure is beneficial for effective ion transportation through the formation of channels in the course of electrochemical processes.^51^

The thermogravimetric analysis (TGA) curve was analysed to ascertain the mass ratio of α-Fe_2_O_3_ to carbon contained in sucrose. The samples were analyzed from ambient temperature to 900 °C at a constant heating rate of 10 °C min^−1^ in air as demonstrated in Fig. S2 (ESI†). It could be observed that bare α-Fe_2_O_3_ maintained a constant profile for the entire period; conversely, 2D C@α-Fe_2_O_3_ begun to loss mass in the range of 100–180 °C and this is designated to the thermal decomposition of the remaining oxygen-associated functional group stack on graphene. The substantial weight loss that occurred within the range of 190–400 °C was due to the oxidation of carbon.^52^ Stability attained after 400 °C indicates that carbon in sucrose has been removed with an estimated mass ratio of 92.85% according to the weight change.

### 3.2. Electrochemical studies

**Fig. 5:**
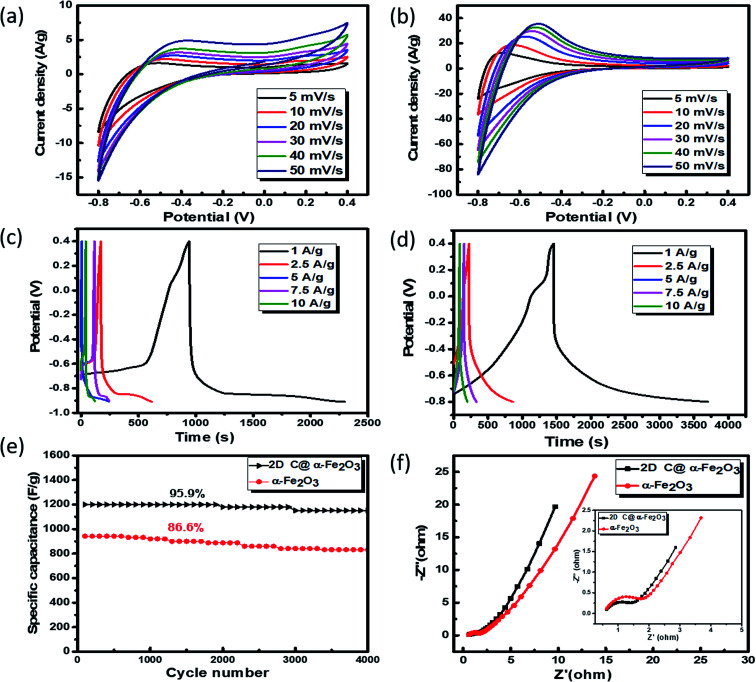
Electrochemical measurements for samples: (a and b) CV curves of α-Fe_2_O_3_ and 2D C@α-Fe_2_O_3_ at different scan rates respectively. GCD profiles at different current densities: (c) α-Fe_2_O_3_ and (d) 2D C@α-Fe_2_O_3_. (e) Cycle performance of 2D C@α-Fe_2_O_3_ and α-Fe_2_O_3_ at a current density of 10 A g^−1^. (f) Nyquist plots of 2D C@α-Fe_2_O_3_ and α-Fe_2_O_3_.

To analyse the electrochemical performance of 2D C @α-Fe_2_O_3_ and α-Fe_2_O_3_, galvanostatic charge/discharge (GCD), cyclic voltammogram (CV) and cyclic stability evaluations were conducted. A wide potential from −0.8 to 0.4 V is selected to ascertain how well the active electrode materials (bare α-Fe_2_O_3_ and 2D C@α-Fe_2_O_3_) could withstand the degradation potential of the aqueous electrolyte medium for anodic and cathodic reactions. Beyond the point of degradation, ionic movement is brought to a halt and the electrolyte begins to react with the system, which is being depicted by the elongated resistive edges in Fig. 5a and b. CV for 2D C@α-Fe_2_O_3_ with a potential window from −0.8 to 0 is shown in Fig. S3a† and clearly, a reduction in the area and resistance is revealed from the curves shown. Fig. 5a and b depicts the CV curves of α-Fe_2_O_3_ and 2D C@α-Fe_2_O_3_ at scan rates from 50 to 5 mV s^−1^. Comparison of the two materials is also shown in Fig. S4a† at a scan rate of 50 mV s^−1^ and the curves shown indicates the faradaic pseudo-capacitance with the CV curve area and redox peaks of 2D C@α-Fe_2_O_3_ was much greater than that of α-Fe_2_O_3_, which probably could be due to cell polarization that came about from hematite electrode resistance. The CV curves shown for the two materials depicted cathodic and anodic pseudocapacitance characteristics of hematite and its carbon-coated ionic particles, which is discernible from the regular rectangular shape associated with the electric double-layer capacitance. The reversible reaction of Fe^2+^ to Fe^3+^ corresponds to the cathodic and anodic redox reaction peaks.^53^ The direct relationship between the peak current and sweep rate undoubtedly ascertain that the electrochemical performance is mainly pivoted on the faradaic redox reaction. It could be observed that there is a linear relationship that exits between the anode peaks and scan rate in the light that the anode peak positions switch more to the anodic order when there is an increase in the scan rate. Furthermore, at high scanning rates, the redox peaks can be maintained, which exhibit the swift electronic and ionic movement processes.^54^Fig. 5c and d shows the charge and discharge profile for α-Fe_2_O_3_ and 2D C@α-Fe_2_O_3_ respectively from a current density of 10 through to 1 A g^−1^. Fig. 5d which represents the measurement for 2D C@α-Fe_2_O_3_ from a current density of 10, 7.5, 5, 2.5, and 1 A g^−1^ had specific capacitance values of 966.7, 1143.75, 1339.58, 1462.5 and 1876.7 F g^−1^. α-Fe_2_O_3_ however in Fig. 5c with the same current density gave specific capacitance values of 845.3, 969.2, 1055.76, 1134.6 and 1142.5 F g^−1^. As clearly observed from the GCD curves, the coulombic efficiencies which describe the charge efficiencies by which dissociated ionic species for conduction are transferred are poor. This is mainly due to the wide potential window (−0.8 to 0.4) selected, which generates a wide stretch reversible reaction between active material's ionic species and the electrolyte. Fig. S3b† shows clearly an improved coulombic efficiency curve for 2D C@α-Fe_2_O_3_ when the potential window is limited to a range of −0.8 to 0. A comparative study of the two materials is shown clearly in Fig. S4b† and all curves showed a fairly good symmetry, but the potential window of α-Fe_2_O_3_ was further extended to −0.9 V while that of 2D C@α-Fe_2_O_3_ ended at 0.8 V. This proves the exceptional performance of 2D C@α-Fe_2_O_3_ judging from the discharge time output and also the shape of the GCD curves, which conforms to that of CV, further substantiating the excellent capacitive behaviour of our samples. The control experiment (CV and GCD) of nickel foam is represented in Fig. S3c and d† to establish the fact that supercapacitive performance observed is primarily assigned to the active electrode material. Fig. S4c† shows a plot of specific capacitance (F g^−1^) against current density pertaining to the two products formed. It shows vividly the outstanding performances 2D C@α-Fe_2_O_3_ over α-Fe_2_O_3_ and the inverse variation that exist between the current density and specific capacitance of our electrode materials. To check the mechanical stability of our samples, cycling performance is measured at a current density of 10 A g^−1^ (Fig. 5e). As demonstrated, the specific capacitance of 2D C@α-Fe_2_O_3_ yielded an appreciable value of 1800 F g^−1^ (95.9% capacity retention) after it had gone through 4000 cycles, while α-Fe_2_O_3_ yielded 982.1 F g^−1^ (86.6 capacity retention) for the same cycle. A comparative study for various synthesis approaches with their ultimate corresponding performance for hematite (α-Fe_2_O_3_) and their respective composites are exhibited in Table 1. It could be clearly observed that the sucrose-assisted microwave combustion method gave the best specific capacitance paralleled to all the other methods with good cycle retention.

**Table 1:** Comparison of electrochemical performance of various synthesis approaches for the preparation of hematite and its composites^a^

Working electrode	Synthesis method	Electrolyte	Potential	Specific capacitance	Cycle life retention	Ref.
α-Fe_2_O_3_	Sol–gel	0.5 M Na_2_SO_3_	−0.8 to 0 V *vs.* SCE	300 F g^−1^ at 1 A g^−1^		56
α-Fe_2_O_3_/rGO/PEDOT: PSS	Self-assembly	1 M KOH	−1.0 to 0 V *vs.* Hg HgO^−1^	875 F g^−1^ at 5 mV s^−1^	100% after 5000 cycles	57
α-Fe_2_O_3_	Electrospinning	1 M LiOH	−0.1 to 0.9 V *vs.* Ag AgCl^−1^	256 F g^−1^ at 1 mV s^−1^	80–82% after 3000 cycles	58
α-Fe_2_O_3_	Anodization	1 M Li_2_SO_4_	−0.8 to 0 V *vs.* SCE	138 F g^−1^ at 1.3 A g^−1^	89% after 500 cycles	59
Fe_2_O_3_ nanodots@N doped G	Solvothermal	2 M KOH	−1.0 to 0 V *vs.* SCE	274 F g^−1^ at 1 A g^−1^	75.3% after 100000 cycle	60
α-Fe_2_O_3_	Wet chemical	1 M H_3_PO_4_	−0.1 to 0.9 V *vs.* Ag AgCl^−1^	308 F g^−1^ at 1 A g^−1^	77% after 1000 cycles	61
α-Fe_2_O_3_/MnO_2_ core–shell	Electrochemical deposition	1 M KOH	−0.1 to (+0.6) V *vs.* Ag AgCl^−1^	838 F g^−1^ at 2 mV s^−1^	98.5% after 1000 cycles	62
α-Fe_2_O_3_ thin film	SILAR method	1 M Na_2_SO_4_	−1.0 to 0 V *vs.* SCE	290 F g^−1^ at 5 mV s^−1^		63
Fe–Ni/Fe_2_O_3_–NiO core/shell hybrid nanostructure	Two-step process	1 M KOH	0–0.55 V *vs.* Ag AgCl^−1^	1415 F g^−1^ at 2.5 A g^−1^	95% after 3000 cycles	64
α-Fe_2_O_3_ on conductive carbon	Hydrothermal	2 M Li_2_SO_4_	−1.0 to 0 V *vs.* Ag AgCl^−1^	1.784 F cm^−2^ at 2 mA cm^−2^		65
Porous Fe_2_O_3_	Template free hydrothermal	1 mol dm^−3^	−0.8 to 0.2 V *vs.* SCE	147 F g^−1^ at 0.36 A g^−1^	86% after 1000 cycles	66
α-Fe_2_O_3_ TF	Spin coating	0.5 M Na_2_SO_3_	−0.8 to 0 V *vs.* Ag AgCl^−1^	365.7 F g^−1^ at 3 A g^−1^		67
α-Fe_2_O_3_ nanomaterials	Solvent mediated precipitation route	0.1 M Na_2_SO_4_	−1.0 to 0.8 V *vs.* Ag AgCl^−1^	450 F g^−1^	88% after 500 cycles	68
α-Fe_2_O_3_	Precipitation	0.1 M Na_2_SO_4_	0–0.8 V *vs.* Ag AgCl^−1^*	200 F g^−1^ at 5 A g^−1^	>99% after 500 cycles	69
α-Fe_2_O_3_ microrods	Chemical treatment	0.5 M Na_2_SO_3_	−1.0 to 0.1 V *vs.* Ag AgCl^−1^	346 F g^−1^ at 2 mV s^−1^	88% after 5000 cycles	70
Graphene foam-CNT@α-Fe_2_O_3_	Atomic layer deposition	2 M KOH	−1.2 to 0 V *vs.* SCE	470.5 mF cm^−2^ at 20 mA cm^−2^	95.4% after 50 000 cycles	71
Fe_2_O_3_/G	One-step chemical reaction	2 mol L^−1^ KOH	−1.0 to 0 V *vs.* SCE	264 F g^−1^ at 2.5 A g^−1^	95.7% after 5000 cycles	72
Fe_2_O_3_ quantum dot/G	Thermal decomposition	1 M Na_2_SO_4_	−1.0 to 0 V *vs.* Ag AgCl^−1^	347 F g^−1^ at 10 mV s^−1^		73
Activated Fe_2_O_3_@carbon core shell structure	Direct current carbon arc discharge	5 M KOH	−1.3 to (−0.3) V *vs.* Hg HgO^−1^	612 F g^−1^ at 0.5 A g^−1^	90% after 10 000 cycles	74
Fe_2_O_3_/ordered mesoporous carbon	Template method	1 M Na_2_SO_3_	−0.8 to 0 V *vs.* Ag AgCl^−1^	677 F g^−1^ at 5 mV s^−1^	89.8% after 1000 cycles	75
Fe_2_O_3_@PPy	Template, hydrothermal, electrochemical polymerization	0.5 M Na_2_SO_4_	−1.0 to (−0.2) V *vs.* SCE	1167.8 F g^−1^ at 1 A g^−1^	97.1% after 3000 cycles	76
Ni(OH)_2_@α-Fe_2_O_3_ core shell structure	Thermal oxidation and hydrothermal method	1 M NaOH	0–0.6 V *vs.* Ag AgCl^−1^	908 F g^−1^ at 21.8 A g^−1^	85.7% after 5000 cycles	77
**2D C@α-Fe**_**2**_ **O**_**3**_	**Sucrose assisted microwave combustion method**	**3 M KOH**	**−0.8 to 0.4 V *vs.* Ag AgCl** ^**−1**^	**1876.7 F g** ^**−1**^ **at 1 A g** ^**−1**^	**95.9% after 4000 cycles**	**This work.**

a Here, TF-thin film; * positive potential range; C-carbon; G-graphene.

Further examination of the electrochemical performance was conducted by equivalent series resistance (EIS) measurement. The equivalent circuit is shown in Fig. S4d,† and the Nyquist plot fit with low and high frequencies is shown in Fig. 5f. A semicircle shape is seen in the high frequency range, which happens to be the charge transfer portion, with a slanted oblique line in the low frequency range depicting the diffusion-limited process. The resistance in series (*R*_S_) which comprises the whole internal resistance such as electrode resistance, resistance from electrolyte and resistance at the interface by contact is measured for the two products. A low *R*_S_ means good faradaic reaction which also implies good conductivity of the electrode material, fast electron transfer rate and short ionic diffusion pathway. *R*_S_ values of 0.977 Ω and 1.192 Ω were calculated for 2D C@α-Fe_2_O_3_ and α-Fe_2_O_3_ respectively, which undoubtedly confirms the spectacular performance of 2D C@α-Fe_2_O_3_.

**Fig. 6:**
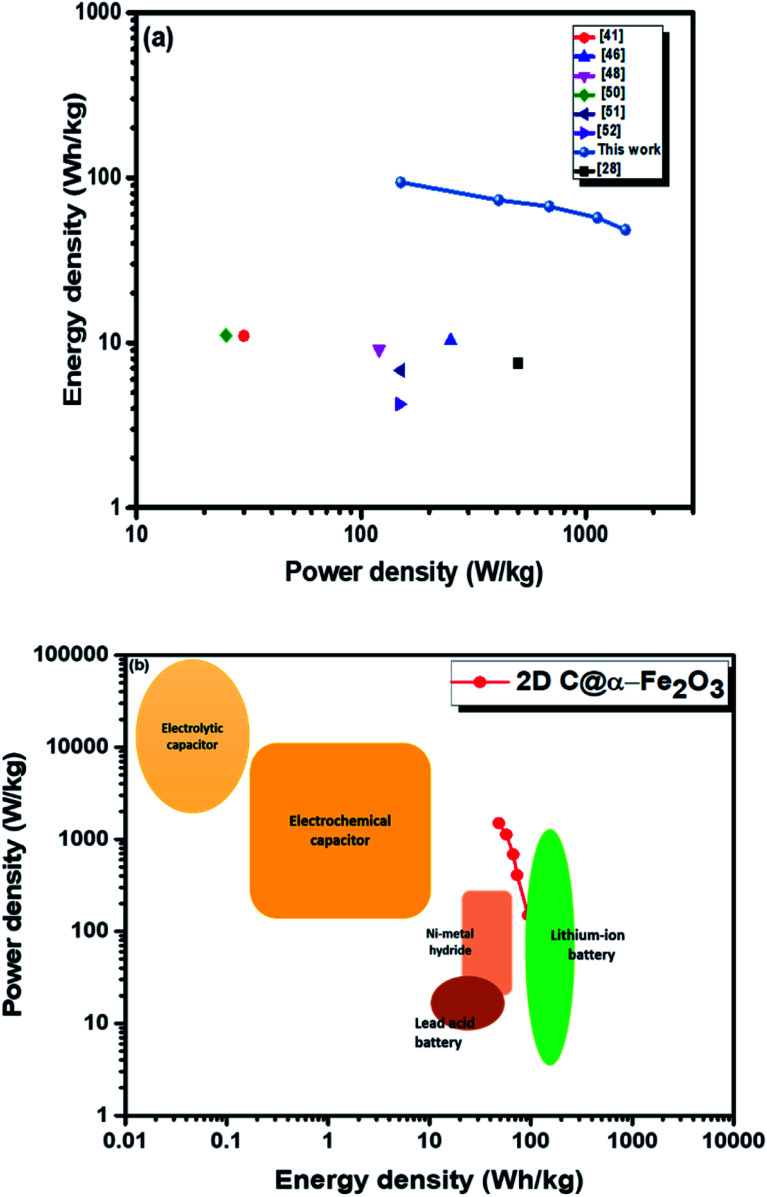
Ragone plots of 2D C@α-Fe_2_O_3_ compared with (a) other reported sucrose- and carbon-based materials and (b) commercial electronic energy storage devices.

Moreover, twice the radius factor of the high frequency region (semicircle portion) represents the charge transfer resistance (*R*_ct_), and it comes about as a result of the faradaic ionic reaction at the electrode–electrolyte interface. Due to this, there is a direct variation between the diameter of the semi-circle and the *R*_ct_ (the smaller the semicircle, the lesser the *R*_ct_ value). From the inset of the graph, it could be clearly seen that 2D C@α-Fe_2_O_3_ has a smaller semicircle diameter as compared to α-Fe_2_O_3_ with *R*_ct_ values of 1.8 Ω and 2.836 Ω correspondingly. Fig. S5 (ESI†) shows a plot of power density against energy density electrode materials. Energy density and power density are measured stationed on the overall mass of both electrodes. It can be evidently observed that both power and energy densities of 2D C@α-Fe_2_O_3_ are much higher than those of pristine α-Fe_2_O_3_. At a power density of 150 W kg^−1^, 2D C@α-Fe_2_O_3_ could attain an ultrahigh energy density of 93.8 W h kg^−1^ while α-Fe_2_O_3_ could attain 57.1 W h kg^−1^ at a power density of 138.46 W kg^−1^. This performance was achieved as a result of the network of interconnected meso/macro-porous structures, which act as traps for the ionic species and easy access of electrolytes to iron particles for good energy storage. The Ragone plots shown in Fig. 6a highlight the performance of our 2D C@α-Fe_2_O_3_ electrode material compared to other carbon-coated hematite electrodes, and Fig. 6b shows the commercial energy storage capacity in which for gravimetric energy storage, the active electrode material constitutes 35–40% of the total packaged mass including binders, collectors and additives.^55^ In both figures, 2D C@α-Fe_2_O_3_ electrode devices undoubtedly presented very good performance comparatively.

## 4. Conclusion

In summary, interconnected meso/macro-porous 2D C@α-Fe_2_O_3_ materials have been synthesized *via* a facile sucrose-assisted microwave combustion route. The macrospores at the surface of the material are well networked to the mesopores within the samples, and this morphological alignment helped to achieve an ultrahigh energy density. The α-Fe_2_O_3_ particles were perfectly coated with an ordered symmetric single-layer graphene in sucrose, which in essence helped improve the performance. Furthermore, addition of sucrose did not entirely alter the morphology and structure of α-Fe_2_O_3_, but improved it and there happen to be a tenacious bond between α-Fe_2_O_3_ and the graphene layer (carbon allotrope) in sucrose. 2D C@α-Fe_2_O_3_ exhibited better electrochemical performance owing to the boost in specific surface area, morphology enhancement and excellent ionic conductivity. 2D C@α-Fe_2_O_3_ showed exceptional mechanical stability with 95.9% capacity retention after 4000 cycles and the Ragone plots vividly show the remarkable energy density attained.

## Conflicts of interest

The authors declare that they have no known competing financial interests or personal relationships that could have appeared to influence the work reported in this paper.

## Supplementary Material

RA-010-D0RA02056G-s001
