# Supplementary material for: Sucrose-templated interconnected meso/macro-porous 2D symmetric graphitic carbon networks as supports for α-Fe2O3 towards improved supercapacitive behavior
Source: RSC Adv. 2020 Apr 21;10(27):15751–62. doi: 10.1039/d0ra02056g (PMC9052401; doi:10.1039/d0ra02056g)
Supplement: RA-010-D0RA02056G-s001 [file RA-010-D0RA02056G-s001.pdf]

Electronic Supplementary Material (ESI) for RSC Advances.
This journal is © The Royal Society of Chemistry 2020

Electronic Supplementary Information

Sucrose templated interconnected meso/macroporous 2D symmetric graphitic carbon network as support for α-Fe_2O_3 towards an improved supercapacitive behavior

Jacob Otabil Bonsu[a], Jeong In Han[b*]

a. Department of Energy and Materials Engineering, Dongguk University – Seoul, Pil-dong, Jung-gu, 04620, Seoul, South Korea.

b. [*]Department of Chemical and Biochemical Engineering, Dongguk University – Seoul, 04620, South Korea.

[*]E-mail: hanji@dongguk.edu

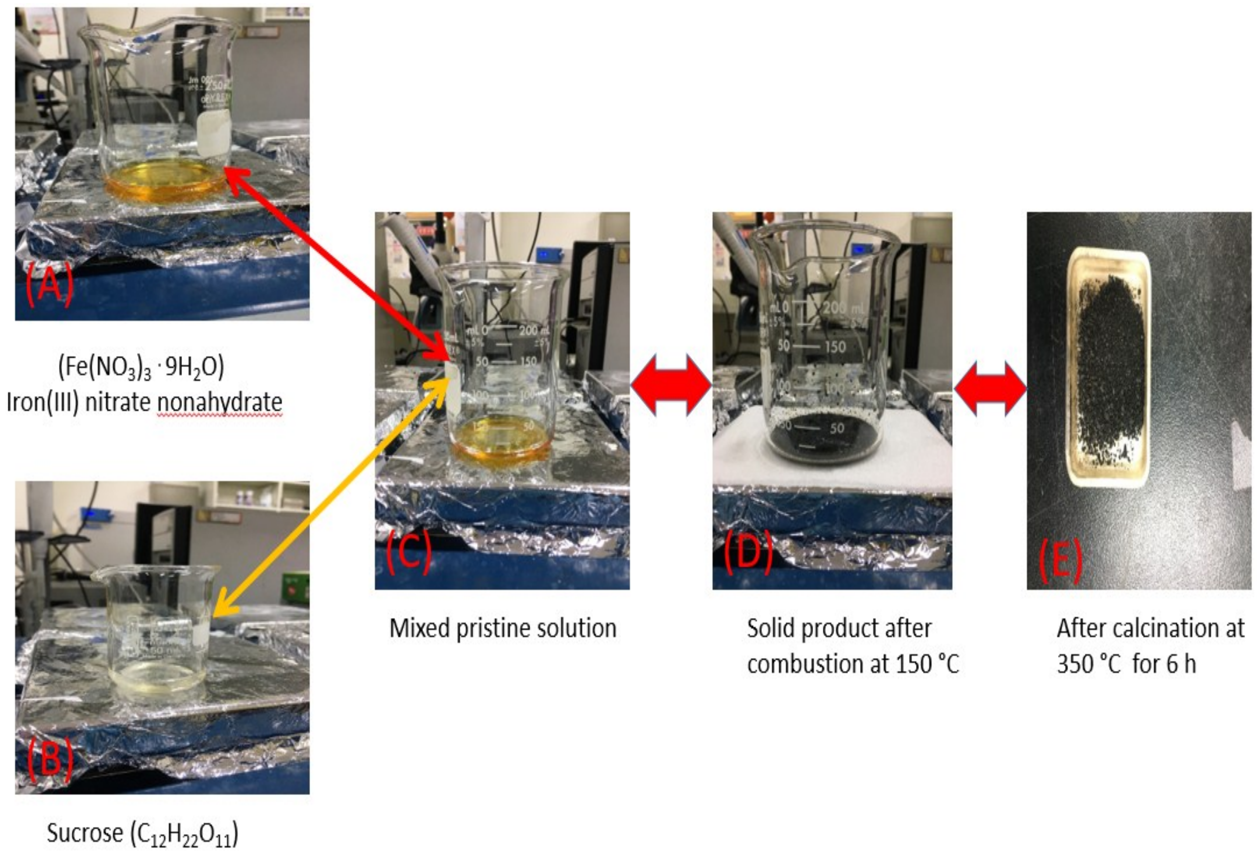

Figure S1: Synthesis procedure for 2D C@ α-Fe_2O_3 and α-Fe_2O_3.

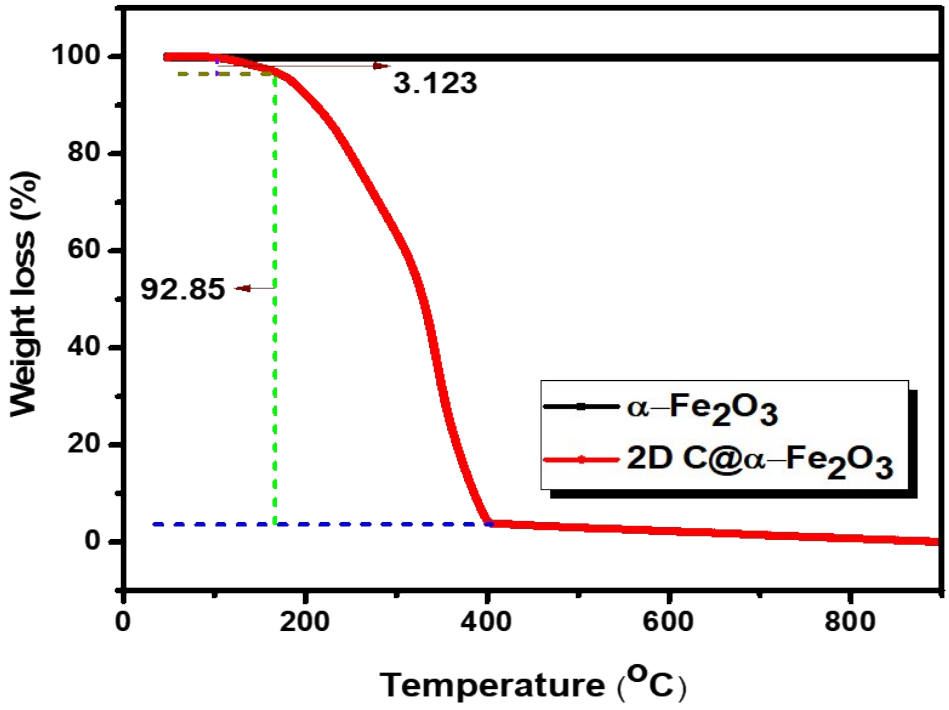

Figure S2: TGA Curves for 2D C@ α-Fe_2O_3 and α-Fe_2O_3 at a constant heating rate of 10 ^{o}C min^{-1}

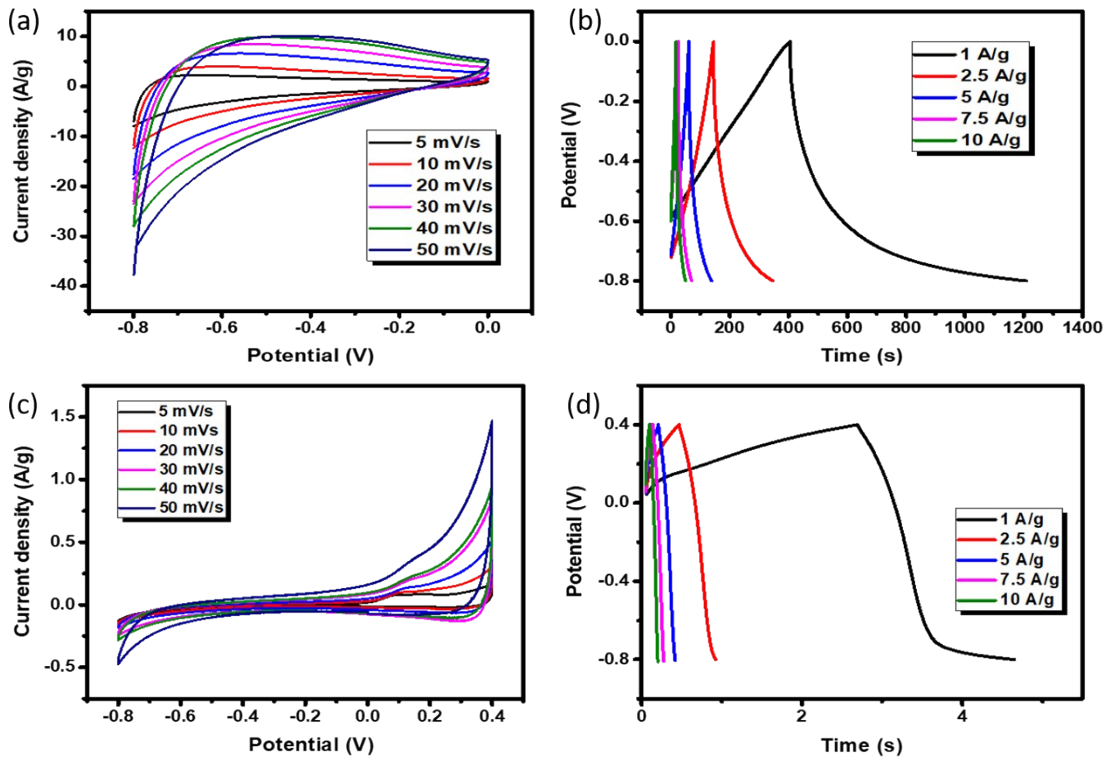

Figure S3: (a) CV for 2D C@ α-Fe_2O_3 with potential window from -0.8~0; (b) GCD for 2D C@ α-Fe_2O_3 with potential window from -0.8~0; (c) CV for Nickel foam; (d) CV for Nickel foam.

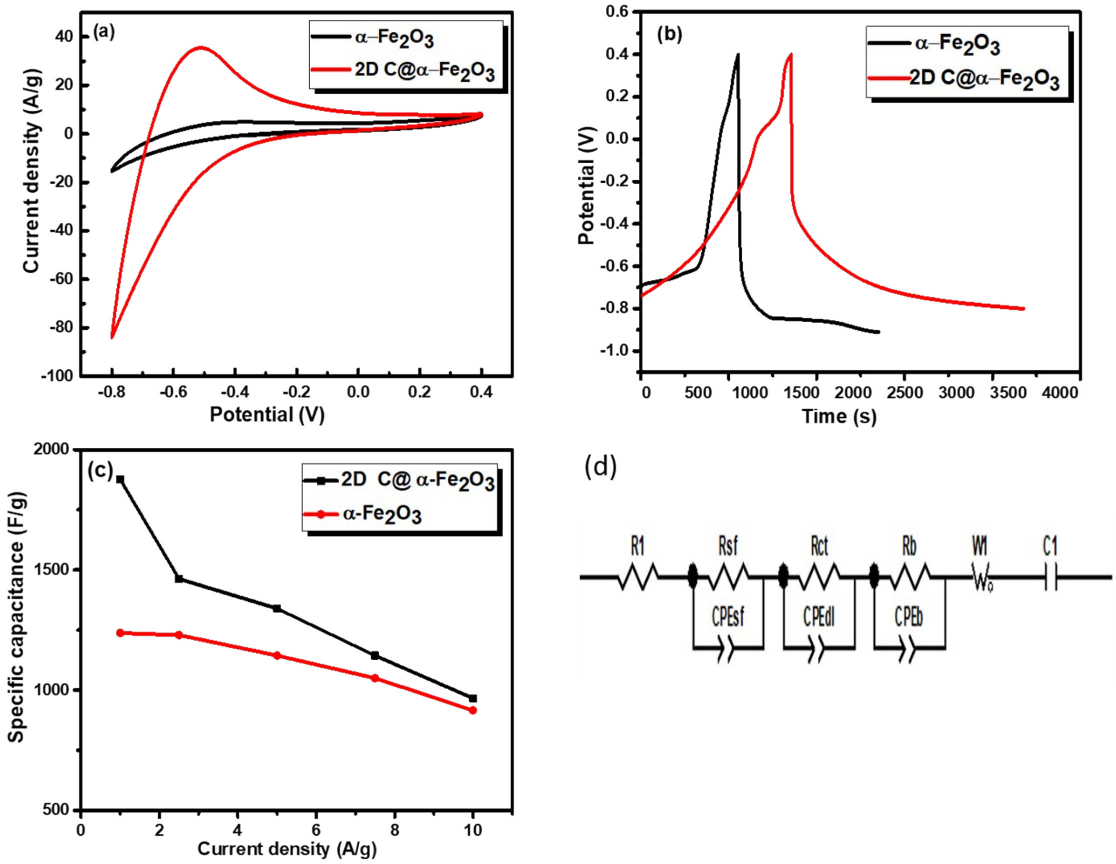

Figure S4: (a) (c) Comparison of CV curves for α-Fe_2O_3 and 2D C@ α-Fe_2O_3 at 50 mV/s scan rate; (b) Comparison of GCD curves for 2D C@ α-Fe_2O_3 and α-Fe_2O_3 at 1 A/g; (c) Plot of specific capacitance as a function of current density; (d) equivalent circuit for hematite.

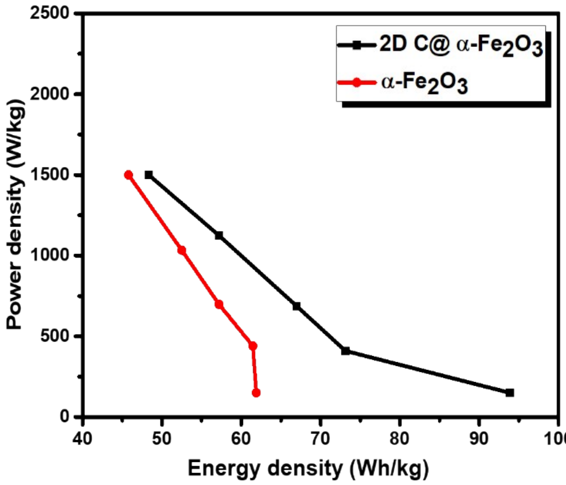

Figure S5: Comparative study for Ragone plot of 2D C@ α-Fe_2O_3 and α-Fe_2O_3.
